# Interactions among common non‐SARS‐CoV‐2 respiratory viruses and influence of the COVID‐19 pandemic on their circulation in New York City

**DOI:** 10.1111/irv.12976

**Published:** 2022-03-12

**Authors:** Haokun Yuan, Alice Yeung, Wan Yang

**Affiliations:** ^1^ Department of Epidemiology, Mailman School of Public Health Columbia University New York New York USA; ^2^ Bureau of Communicable Disease New York City Department of Health and Mental Hygiene New York New York USA

**Keywords:** coronaviruses, COVID‐19 pandemic impact, human metapneumovirus, parainfluenza, respiratory virus interactions, rhinovirus

## Abstract

**Background:**

Non‐pharmaceutical interventions (NPIs) and voluntary behavioral changes during the COVID‐19 pandemic have influenced the circulation of non‐SARS‐CoV‐2 respiratory infections. We aimed to examine interactions among common non‐SARS‐CoV‐2 respiratory virus and further estimate the impact of the COVID‐19 pandemic on these viruses.

**Methods:**

We analyzed incidence data for seven groups of respiratory viruses in New York City (NYC) during October 2015 to May 2021 (i.e., before and during the COVID‐19 pandemic). We first used elastic net regression to identify potential virus interactions and further examined the robustness of the found interactions by comparing the performance of Seasonal Auto Regressive Integrated Moving Average (SARIMA) models with and without the interactions. We then used the models to compute counterfactual estimates of cumulative incidence and estimate the reduction during the COVID‐19 pandemic period from March 2020 to May 2021, for each virus.

**Results:**

We identified potential interactions for three endemic human coronaviruses (CoV‐NL63, CoV‐HKU, and CoV‐OC43), parainfluenza (PIV)‐1, rhinovirus, and respiratory syncytial virus (RSV). We found significant reductions (by ~70–90%) in cumulative incidence of CoV‐OC43, CoV‐229E, human metapneumovirus, PIV‐2, PIV‐4, RSV, and influenza virus during the COVID‐19 pandemic. In contrast, the circulation of adenovirus and rhinovirus was less affected.

**Conclusions:**

Circulation of several respiratory viruses has been low during the COVID‐19 pandemic, which may lead to increased population susceptibility. It is thus important to enhance monitoring of these viruses and promptly enact measures to mitigate their health impacts (e.g., influenza vaccination campaign and hospital infection prevention) as societies resume normal activities.

## 1 INTRODUCTION

Viral respiratory infections are one of the leading causes of disease in humans. Each year, numerous respiratory viruses co‐circulate in the population, causing substantial public health burden^1^ and economic loss.^2^, ^3^ Previous studies have suggested that respiratory viruses may interfere with and change the risk, timing, or natural history of infection of one another.^4^ For instance, in 2009, seasonal epidemic of respiratory syncytial virus (RSV) in Israel was temporarily delayed due to the A(H1N1) pandemic.^5^ Potential mechanisms including competitions within hosts (e.g., infecting cells) and population‐level interactions have been proposed to explain such virus interactions.^4^ However, the specific interactions among different respiratory viruses and the impact on their collective transmission dynamics have not been well characterized.

Before the COVID‐19 pandemic, influenza was the foremost public health concern among all respiratory infections. As such, much research effort has been devoted to understand the transmission dynamics of influenza viruses and their interactions with other respiratory viruses.^4^ In contrast, many other infections such as human endemic coronaviruses have received far less attention. In addition, previous studies tend to ignore the different subtypes of respiratory viruses and only examine interactions at the level of genus. However, subtypes from the same virus group may have different seasonality (e.g., the four subtypes of parainfluenza viruses) and competitions within genus tend to be more intense (e.g., influenza A[H1N1] and A[H3N2]).^6^ As such, combining all subtypes of a virus regardless of their circulation patterns may mask the true interactions.

Following its emergence in late 2019, the severe acute respiratory syndrome coronavirus 2 (SARS‐CoV‐2) has spread to 214 countries and territories, causing the coronavirus disease 2019 (COVID‐19) pandemic.^7^ The widespread prevalence of SARS‐CoV‐2 may affect the circulation of other respiratory viruses, via virus interactions. In addition, during the COVID‐19 pandemic, non‐pharmaceutical interventions (NPIs) such as social distancing, school closure, travel ban, and mask‐wearing that aimed to mitigate SARS‐CoV‐2 transmission had also limited the transmission of other respiratory viruses. Seasonal respiratory viruses such as influenza were found to be at low circulation during the 2020 respiratory virus season amid the COVID‐19 pandemic.^8^, ^9^ However, to what extent the COVID‐19 pandemic has impacted the circulation of common respiratory viruses and potential differences by virus remains unclear.

In this work, we analyzed incidence data for seven groups of respiratory viruses in New York City (NYC) before and during the COVID‐19 pandemic. We first used elastic net regression to identify potential interactions among the viruses at the subtype level (13 in total). We further hypothesized that strong interactions should allow models incorporating the relationship to more accurately predict the incidence of related respiratory viruses. To examine the robustness of the found interactions, we thus built and compared the performance of Seasonal Auto Regressive Integrated Moving Average (SARIMA) models with and without the interactions. Lastly, we used the best‐performing models to estimate the impact of the COVID‐19 pandemic on circulation of each of the 13 respiratory viruses.

## 2 METHODS

### 2.1 Virus surveillance data

The virus surveillance data were collected from a subset of NYC participating laboratories that test for multiple respiratory viruses in addition to influenza and RSV. Tested viruses included adenovirus (Adv), coronavirus (CoV), human metapneumovirus (HMPV), rhinovirus (RV), parainfluenza (PIV), RSV, and influenza virus (IV) overall and by subtype (Table 1). The data included the number of respiratory pathogen panel tests requested each week and the number tested positive for each virus during Week 40 of 2015 to Week 20 of 2021. Prior to the COVID‐19 pandemic, the total number of samples tested each respiratory virus season (defined as the 40th week of the year to the 39th week the next year) increased over time with the expansion of testing (Figure S1). Previous studies have used the percent positivity (i.e., the ratio of positive samples to total samples) to account for such changes in testing. However, during the summer when fewer respiratory viruses are in circulation and fewer people seek testing, the much smaller sample sizes (Figure S1) tend to result in very high percent positivity for some viruses (e.g., RV in Figure S2), which may not reflect the true circulation levels of these viruses in the population. Thus, to account for the testing time trend and better represent the viral circulation levels, here, we adjusted the weekly incidence by multiplying the ratio of the number of samples tested during the season to that number in season 2015–2016 (the first season of data collection). For the COVID‐19 pandemic period (Week 10 of 2020 to Week 20 of 2021; note the first COVID‐19 case was reported in NYC during Week 10 of 2020^10^), the number of samples tested each week fluctuated substantially from week to week (in particular, there were initial increases followed by a large drop during early weeks of the COVID‐19 pandemic period; see Figure S1). To account for this short‐term fluctuation, we adjusted the incidence during COVID‐19 pandemic period week by week relative to the corresponding week during season 2015–2016. A comparison of the adjusted weekly incidence using this method and the percent positivity for each virus is shown in Figure S2.

**TABLE 1:** Model‐identified potential viral interactions during the pre‐COVID period

Respiratory virus (sub)type		Virus interactions	Out‐of‐fit model validation: Observed incidence vs. out‐of‐fit estimates (week 40 of 2019 – Week 9 of 2020)		
			Observed	Estimated (SARIMA)	Estimated (SARIMAX)
Adenovirus (Adv)		IV, RSV, CoV‐NL63, RV, HMPV	416.27	518 (190, 862)	601 (274, 934)
Human endemic coronavirus (CoV)	CoV‐NL63	CoV‐OC43	314.65	493 (205, 846)	475 (193, 825)
	CoV‐HKU	IV, CoV‐NL63	553.51	470 (315, 678)	442 (303, 626)
	CoV‐OC43	CoV‐229E, RV, IV, Adv, CoV‐NL63, HMPV, RSV	138.74	269 (77, 557)	269 (69, 529)
	CoV‐229E	CoV‐OC43, PIV‐2	43.03	15 (2, 180)	6 (2, 138)
Human Metapneumovirus (HMPV)		IV, PIV‐3, CoV‐OC43	271.15	373 (123, 671)	439 (221, 677)
Rhinovirus (RV)		PIV‐2, PIV‐3, Adv, PIV‐4, CoV‐OC43, CoV‐NL63	2484.9	2664 (1097, 4231)	2105 (824, 3455)
Parainfluenza (PIV)	PIV‐1	RV, Adv	179.01	120 (25, 250)	126 (39, 247)
	PIV‐2	RV	17.27	15 (2, 134)	17 (2, 132)
	PIV‐3	RV, PIV‐4, CoV‐229E, CoV‐HKU, HMPV, IV, CoV‐OC43, PIV‐1	63.96	118 (2, 693)	31 (2, 450)
	PIV‐4	CoV‐NL63, PIV‐2, RSV	177.08	90 (7, 254)	64 (3, 229)
Respiratory Syncytial Virus (RSV)		CoV‐OC43, Adv, RV, IV, PIV‐4	1242.7	1545 (879, 2257)	1416 (820, 2049)
Influenza virus (IV)		HMPV, Adv	2024.45	1540 (213, 4070)	1422 (196, 3812)

*Note*: Column “virus interactions” show identified interactions from the initial selection by the elastic net regression and the stepwise forward selection for the SARIMAX model (i.e., these were the final variables included in the SARIMAX model). Estimated strengths of interactions are shown in Table S2. The third panel shows comparison between the observed cumulative incidence and model out‐of‐fit estimates (mean and prediction intervals in parentheses) during the testing period (i.e., Week 40 of 2019 to Week 9 of 2020). In this model validation, the prediction intervals included the observed value for all model out‐of‐fit estimates; thus, all were deemed accurate.

### 2.2 Selection of key viral interactions during the pre‐COVID period

We used elastic net regression models^11^ to preliminarily identify, for each virus, the set of other viruses consistently included as interacting viruses, during Week 40 of 2015 to Week 9 of 2020, that is, before the first COVID‐19 case was reported in NYC.^10^ To avoid spurious correlation due to a common winter‐time seasonality shared by some viruses,^12^ we first used a linear regression model to identify and remove the seasonal trend for each virus. The model took the following form:

(1) Y∼Week_of_Year,

where *Y* is the adjusted weekly incidence (see Section 2.1) and *Week_of_Year* is an indicator variable for each week of the calendar year (1:52 for annual seasonal cycle and 1:104 for biennial cycles). For each virus, we fitted both annual and biennial cycles and used the adjusted *R* ^2^ to determine the most likely seasonal cycle for each virus for removal of seasonal trend.

We then regressed on the detrended time series (i.e., the residuals after removing the seasonal trend) with an elastic net penalty:

(2) $$Y_{i,\text{detrended}}=\sum_{j\neq i}\beta_{j}X_{j,\text{detrended}},$$

where *Y*_*i, detrended*_ is the detrended weekly incidence for a given virus of interest, *i*, and *X*_*j, detrended*_ is the detrended weekly incidence for other viruses (i.e., for any *j* ≠ *i*); $\beta_{j}$ is the corresponding regression coefficient. Similar to lasso (i.e., least absolute shrinkage and selection operator) and ridge regressions, elastic net shrinks regression coefficients by imposing a penalty on their size. However, instead of penalizing by the sum of absolute coefficients (L1—lasso penalty) or the sum‐of‐squares coefficients (L2—ridge penalty), elastic net regression penalizes with both L1 and L2 and the penalty function is formulated as

(3) $$\lambda \sum_{j=1}^{p}\left(\alpha \beta_{j}^{2}+\left(1-\alpha \right)\left|\beta_{j}\right|\right),$$

where λ controls the amount of shrinkage, α controls the distribution between L1 and L2, and $\beta_{j}$ represents the regression coefficients that minimize the penalty (i.e., formula 3). Since elastic net shares traits of both ridge and lasso regression, while it selects covariates like lasso, it also allows coefficients of correlated covariates to shrink together and provide a more stable selection result.

We fitted 500 elastic net regressions with tenfold cross‐validation and pooled all interactions selected at least in half of the 500 runs for further testing (see the next section). Thirteen models were developed, one model each for Adv, HMPV, RV, RSV, and IV and for each of four subtypes of CoV and PIV. For influenza, due to the more erratic circulation pattern of different types and subtypes and short study period with available data (i.e., 5 years), we combined all types and subtypes.

### 2.3 Testing the identified interactions

To further test the identified interactions from the elastic net regression models, we examined if inclusion of any of the found interactions in an SARIMA model with exogenous variables (SARIMAX) model^13^ improves model fit, compared to an SARIMA model. Here, we used seasonal models because a seasonal trend was identified for all viruses using Equation 1. For the seasonal component, we applied seasonal differencing once to each incidence time series with the same seasonal period as the identified seasonal cycle from Equation 1; for simplicity, we did not include any seasonal autoregressive or seasonal moving average terms. After the seasonal differencing, the SARIMA model took the following form:

(4) $$y_{t}^{\prime }=\sum_{i=1}^{p}\phi_{i}y_{t-i}^{\prime }+\sum_{i=1}^{q}\theta_{i}\varepsilon_{t-i}+\varepsilon_{t},$$

where $y^{\prime }$ is a differenced time series with a seasonal differencing degree of 1 and non‐seasonal differencing degree *d*; *p* is the order of autoregressive (AR) model; *q* is the order of moving average (MA) model; $\phi_{i}$ (*i* = 1, …, *p*) are the coefficients for the AR terms; $\theta_{i}\;\left(i=1,\ldots q\right)$ are the coefficients for the MA terms; and ε is the error term. Parameters *d*, *p*, and *q* were selected based on model fit, and the values from the best‐fit model for each virus are shown in Table S1.

Similarly, after the seasonal differencing, the SARIMAX model was formulated as

(5) $$y_{t}^{\prime }=\sum_{i=1}^{r}\beta_{i}x_{i,t}+\sum_{i=1}^{p}\phi_{i}y_{t-i}^{\prime }+\sum_{i=1}^{q}\theta_{i}\varepsilon_{t-i}+\varepsilon_{t},$$

where $x_{i}\;\left(i=1,\ldots ,r\right)$ are the exogenous variables (here, virus interactions identified by the elastic net regression) and $\beta_{i}\;\left(i=1,\ldots ,r\right)$ the corresponding coefficients. For each virus, we set *d*, *p*, and *q* to the same values as the best‐fit SARIMA model (Table S1) and used forward stepwise selection to identify the exogenous variables (i.e., virus interactions). That is, virus interactions that do not improve model fit, based on the Akaike information criterion (AIC),^14^ are excluded in this step. Viruses included in the SARIMAX model with the lowest AIC are identified as potential interactions and presented in Table 1.

### 2.4 Model validation

We tested the SARIMA and SARIMAX models using data prior to the COVID‐19 pandemic (i.e., Week 40 of 2015 to Week 9 of 2020). Specifically, we divided this pre‐COVID dataset into a training (Week 40 of 2015 to Week 39 of 2019, i.e., four full respiratory seasons) and a testing (Week 40 of 2019 to Week 9 of 2020, i.e., the last respiratory season before the COVID‐19 pandemic) subset. The models (either SARIMA or SARIMAX) were first fit to the training subset; the trained models were then used to estimate the weekly incidence for each virus during the testing period for out‐of‐fit model validation. We compared model performance based on relative Root Mean Square Error (rRMSE) during the testing period. In addition, as the weekly incidence tended to be low, we also evaluated the models based on the cumulative incidence during the testing period and the 95% prediction interval; if the observed cumulative incidence fell within the 95% prediction interval, the model was deemed accurate.

### 2.5 Estimating the impact of COVID‐19 pandemic on circulation of non‐SARS‐CoV‐2 viruses

We used the validated models (SARIMA or SARMIAX) to generate counterfactual estimates for each virus during the pandemic period—that is, the expected cumulative incidence should there be no pandemic. To enhance model performance, we refitted the validated models using data during the entire pre‐COVID period (i.e., through Week 9 of 2020) and used them to predict the incidence for each virus during the COVID‐19 period (here Week 10 of 2020 to Week 20 of 2021). We then compared the model counterfactual estimates of cumulative incidence during the COVID‐19 period (*C*_*counterfactual*_) to the observations (*C*_*observed*_) to estimate the impact of the COVID‐19 pandemic. We computed the percent reduction in cumulative incidence due to the COVID‐19 pandemic for each virus as

(6) $$\text{reduction}\;\left(\%\right)=\frac{C_{\text{observed}}-C_{\text{counterfactual}}}{C_{\text{counterfactual}}}\times 100\%.$$

## 3 RESULTS

### 3.1 Respiratory virus circulation before and after the introduction of SARS‐CoV‐2

**FIGURE 1:**
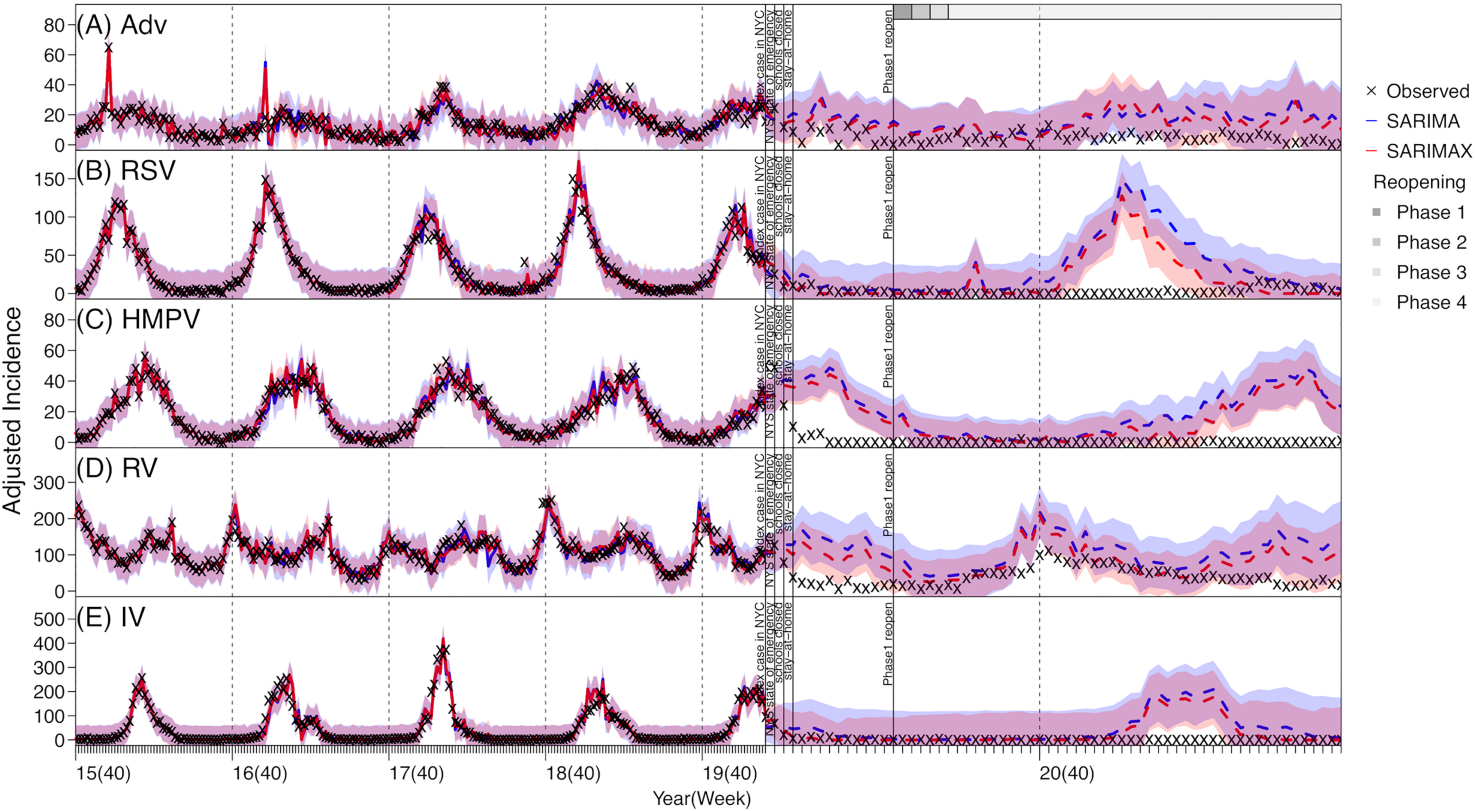
Model fit and counterfactual‐estimates of weekly incidence for adenovirus (Adv; A), respiratory syncytial virus (RSV; B), human metapneumovirus (HMPV; C), rhinovirus (RV; D), and influenza virus (IV; E) using SARIMA and SARIMAX models. Crosses (“x”) show scaled weekly incidence. Blue lines (mean) and shaded areas (95% confidence intervals) show model fit (solid lines) and counterfactual‐estimates (dashed lines) using the SARIMA models; red lines (mean) and shaded areas (95% confidence intervals) show model fit (solid lines) and counterfactual‐estimates (dashed lines) using the SARIMAX models. Vertical dashed lines indicate the start of each respiratory virus season, and vertical black lines mark timing of major COVID‐19 events; gray bars on the top of the plot indicate different reopening phases in NYC (see criteria of reopening and phases at https://forward.ny.gov). Note the *x*‐axis scale for the pandemic counterfactual‐estimates is expanded to show more details

**FIGURE 2:**
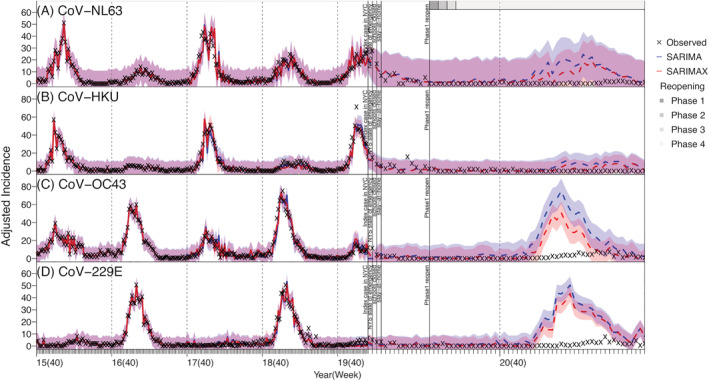
Model fit and counterfactual‐estimates of weekly incidence for human endemic coronaviruses: Cov‐NL63 (A), Cov‐HKU (B), Cov‐OC43 (C), and Cov‐229E (D) using SARIMA and SARIMAX models. Blue lines (mean) and shaded areas (95% confidence intervals) show model fit (solid lines) and counterfactual‐estimates (dashed lines) using the SARIMA models; red lines (mean) and shaded areas (95% confidence intervals) show model fit (solid lines) and counterfactual‐estimates (dashed lines) using the SARIMAX models. Vertical dashed lines indicate the start of each respiratory virus season, and vertical black lines mark timing of major COVID‐19 events; gray bars on the top of the plot indicate different reopening phases in NYC (see criteria of reopening and phases at https://forward.ny.gov). Note the *x*‐axis scale for the pandemic counterfactual‐estimates is expanded to show more details

**FIGURE 3:**
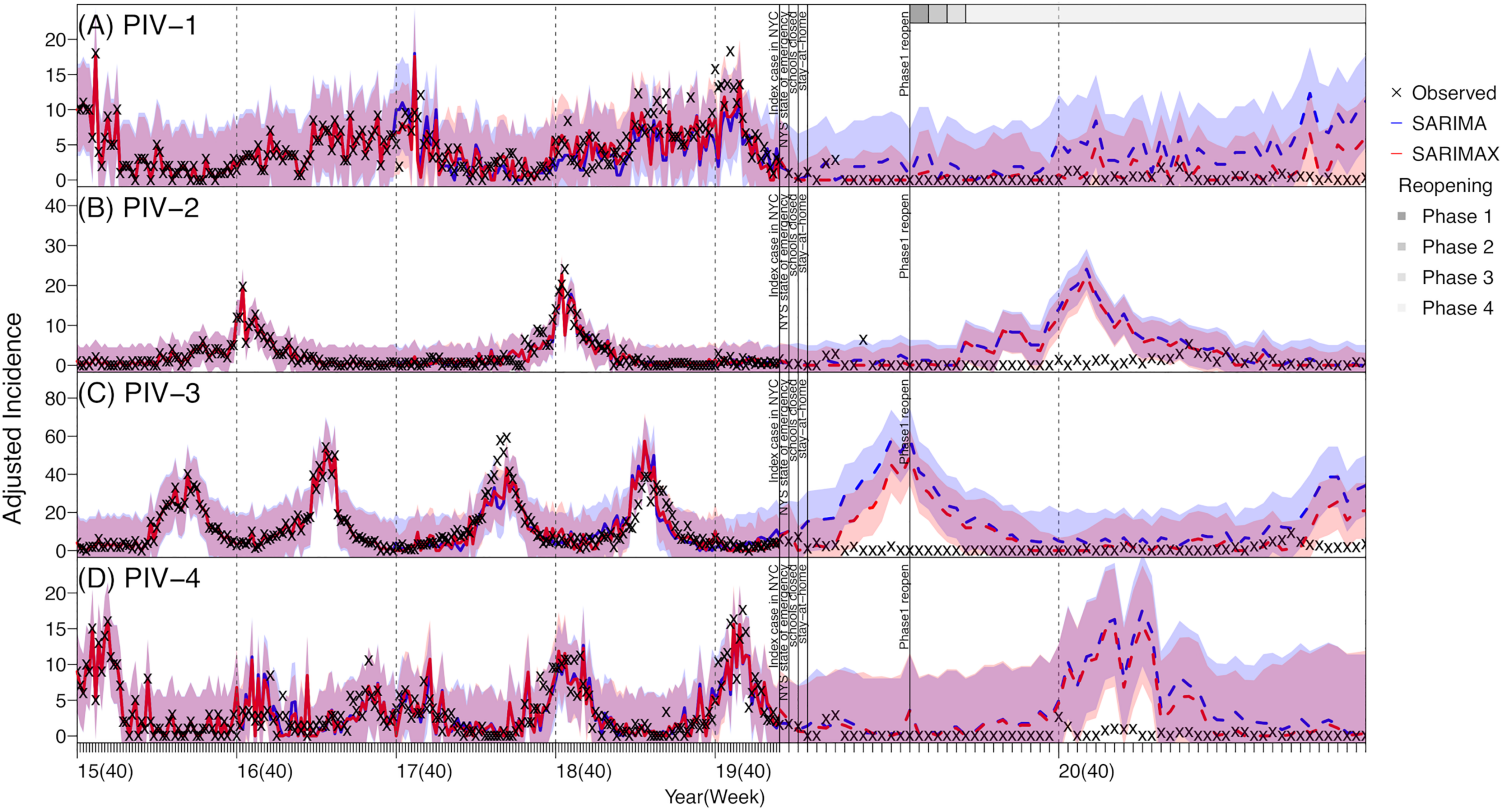
Model fit and counterfactual‐estimates of weekly incidence for parainfluenza viruses: PIV‐1 (A), PIV‐2 (B), PIV‐3 (C), and PIV‐4 (D) using SARIMA and SARIMAX models. Blue lines (mean) and shaded areas (95% confidence intervals) show model fit (solid lines) and counterfactual‐estimates (dashed lines) using the SARIMA models; red lines (mean) and shaded areas (95% confidence intervals) show model fit (solid lines) and counterfactual‐estimates (dashed lines) using the SARIMAX models. Vertical dashed lines indicate the start of each respiratory virus season, and vertical black lines mark timing of major COVID‐19 events; gray bars on the top of the plot indicate different reopening phases in NYC. (see criteria of reopening and phases at https://forward.ny.gov). Note the *x*‐axis scale for the pandemic counterfactual‐estimates is expanded to show more details

During the pre‐COVID period, influenza viruses were the most commonly detected in our dataset (up to 400 adjusted case counts per week), followed by RV (up to ~250 per week), CoV and RSV (both up to ~150 per week); in comparison, other viruses (HMPV, Adv, and PIV) tended to have low cases detected (around 40–60 cases during peak weeks; Figure S2). Most of the respiratory viruses (IV, Adv, CoV, RSV, and HMPV) included here had outbreaks in the winter every year (Figures 1 and 2). In contrast, RV cases were detected year‐round and tended to have two comparable epidemics each year—one in the winter and one in the summer (Figure 1). PIV cases were also detected throughout the year, but the outbreak patterns were less obvious (Figure 3). For the same virus group, different subtypes tended to alternate in circulation and recurred biennially with irregular peaks (e.g., the four coronaviruses in Figure 2; PIV‐1 and PIV‐2 in Figure 3). In addition, among the four coronaviruses, weekly case counts were highly correlated for virus pairs belonging to different genera (*r* = 0.82 between CoV‐OC43 and CoV‐229E and 0.76 between CoV‐NL63 and CoV‐HKU). In contrast, the circulation of different influenza types and subtypes and PIV‐4 appeared less regular, with few cases detected in some years.

### 3.2 Potential virus interactions

The elastic net regression combining with the SARIMAX model selection identified several potential associations among the respiratory viruses (Tables 1 and S2). However, the patterns of found interactions were not readily clear (Table S2). Overall, found interactions appeared stronger and more robust (e.g., bidirectional interaction for both involved viruses) for virus‐pairs from the same virus type. For instance, the models estimated that there were positive, bidirectional interactions between CoV‐NL63 and CoV‐OC43 and between CoV‐229E and CoV‐OC43. This likely reflects the stronger interactions among closely related viruses. In addition, the models identified potential positive interactions among two viruses (RV and Adv) that tended to circulate year‐round and three PIVs (PIV‐1, PIV‐2, and PIV‐3) that tended to circulate during the summer and/or early fall (Table S2 and Figure S2).

Further examination using the time series models indicated that, for three coronaviruses (i.e., CoV‐NL63, CoV‐HKU, and CoV‐OC43), RV, PIV‐1, and RSV, the inclusion of the identified interacting viruses in the SARIMAX model generated more accurate out‐of‐fit estimates than the SARIMA model (Table S3). However, this improvement was not substantial (2.31–15.85% reduction in rRMSE, see Table S3), likely because epidemics of these respiratory viruses were strongly driven by their underlying seasonality. For the remaining viruses, the inclusion of the interactions did not improve the performance of the SARIMAX model. Overall, out‐of‐fit estimates of weekly incidence (Figures [Link], [Link], [Link]) and cumulative incidence during the testing period (Week 40 of 2019 to Week 9 of 2020, i.e., the main part of the last respiratory virus season before the COVID‐19 pandemic; Table 1) were similarly accurate for both time series models. As such, below we present results from both models.

### 3.3 Impact of the COVID‐19 pandemic on non‐SARS‐CoV‐2 viruses

Most respiratory viruses included here appeared to have had lower circulation during the COVID‐19 pandemic than would be expected (comparing the observed incidence and model counterfactual estimates in Figures 1, 2, 3). This is likely due to the continued NPIs implemented in NYC during March 2020 to May 2021 (the end of our study period; see Figures 1, 2, 3 for major NPIs implemented). In particular, the circulation of CoV‐OC43, CoV‐229E, HMPV, PIV‐2, PIV‐4, RSV, and IV all reduced significantly during the COVID‐19 pandemic period—the observed cumulative incidence during this period fell outside the model predicted 95% intervals and the estimated mean reduction was around 70–90% for these viruses (Table 2). In contrast, Adv and RV appeared to be less affected (Figure 1A,D). Incidence of these two viruses, while low, was nonzero during most weeks of the pandemic period (59 and 63 out of 63 weeks, respectively), and for RV, incidence increased substantially during the summer of 2020, despite the implemented NPIs (Figure 2D). For CoV‐NL63 and CoV‐HKU, the models did not detect a significant reduction, likely because epidemics of these viruses tended to recur every other year and their lull periods coincided with the pandemic period (Figure 2A,B).

**TABLE 2:** Estimated impact of the COVID‐19 pandemic on circulation of non‐SARS‐CoV‐2 viruses in NYC

Respiratory virus (sub)type		Cumulative incidence during the COVID‐19 period				
		Observed	SARIMA model counterfactual estimates		SARIMAX model counterfactual estimates	
			Modeled	% reduction	Modeled	% reduction
Adenovirus (Adv)		360.33	1,061 (146, 2,185)	−66% (−84%, 147%)	930 (114, 2,056)	−61% (−82%, 216%)
Human endemic coronavirus (CoV)	CoV‐NL63	148.49	495 (25, 1,679)	−70% (−91%, 494%)	363 (16, 1,516)	−59% (−90%, 828%)
	CoV‐HKU	124.09	246 (4, 975)	−50% (−87%, 3,002%)	165 (7, 820)	−25% (−85%, 1,673%)
	CoV‐OC43	132.53	921 (505, 1,790)	**−86% (−93%, −74%)**	504 (239, 1,247)	**−74% (−89%, −45%)**
	CoV‐229E	78.36	675 (431, 1,125)	**−88% (−93%, −82%)**	535 (336, 950)	**−85% (−92%, −77%)**
Human Metapneumovirus (HMPV)		154.72	1,278 (591, 2,197)	**−88% (−93%, −74%)**	1,051 (512, 1,829)	**−85% (−92%, −70%)**
Rhinovirus (RV)		2709.14	7,045 (2,472, 11,837)	−62% (−77%, 10%)	5414 (1,754, 9,398)	−50% (−71%, 54%)
Parainfluenza (PIV)	PIV‐1	23.51	227 (23, 647)	−90% (−96%, 2%)	68 (3, 396)	−65% (−94%, 684%)
	PIV‐2	52.05	290 (122, 614)	**−82% (−92%, −57%)**	242 (105, 534)	**−78% (−90%, −50%)**
	PIV‐3	85.25	862 (78, 2,558)	−90% (−97%, 9%)	232 (2, 1,418)	−63% (−94%, 4,162%)
	PIV‐4	20.43	237 (50, 762)	**−91% (−97%, −59%)**	189 (37, 735)	**−89% (−97%, −45%)**
Respiratory Syncytial Virus (RSV)		250.3	1,814 (561, 4,205)	**−86% (−94%, −55%)**	1,453 (519, 3,507)	**−83% (−93%, −52%)**
Influenza (IV)		258.09	2,924 (584, 10,831)	**−91% (−98%, −56%)**	1,941 (424, 9,033)	**−87% (−97%, −39%)**

*Note*: Column “Observed” shows the scaled cumulative incidence as recorded during the COVID‐19 period (March 2020 to May 2021) for each virus (listed in column “Respiratory Virus (Sub)type”). The “Modeled” columns show model‐counterfactual estimates (mean and 95% prediction interval in parentheses) using the SARIMA or SARIMAX model (specified in the row above), and the “% Reduction” columns show estimated percent reduction (mean and 95% confidence interval) during the COVID‐19 pandemic period, per Equation 6. Significant reductions are bolded.

## 4 DISCUSSION

In this study, we utilized viral surveillance data collected in NYC before and during the COVID‐19 pandemic to examine potential interactions among seven groups of respiratory viruses and the impact of the COVID‐19 pandemic on their circulation in the population. We identified several potential interactions, particularly, for three coronaviruses (CoV‐NL63, CoV‐HKU, and CoV‐OC43), PIV‐1, RV, and RSV. In addition, we found a significantly lower number of cases were detected for several viruses (i.e., CoV‐OC43, CoV‐229E, HMPV, PIV‐2, PIV‐4, RSV, and IV) in 2020–2021, suggesting reduced circulation of these viruses, during the COVID‐19 pandemic. Consistently, other studies have also found that circulation of influenza, seasonal coronaviruses, RSV, PIV, and HMPV reduced substantially in the United States^8^ and Canada^15^ and that RV was less affected,^8^, ^15^ during 2020–2021.

Our analysis found multiple potential interactions among the 13 viruses and that most associations were positive, before the COVID‐19 pandemic (Table S2). While these findings are based on population‐level data and cannot be interpreted directly as viral interference, the identified associations appear to in part reflect the underlying viral interactions. In particular, we note two factors that may have contributed to the found positive associations. First, for viruses of the same group, stronger competition between those within the same genus may reduce their chance to cocirculate and indirectly result in co‐circulation of the ones from different genera (e.g., the estimated positive associations between the beta‐coronavirus CoV‐OC43 and the two alpha‐coronaviruses CoV‐NL63 and CoV‐229E, separately). Second, many viral infections share similar transmission routes and/or environmental factors^16^ and, as a result, are more likely to occur around the same time. For instance, here, we found positive associations between RV and three PIVs (i.e., PIV 1–3), all of which could circulate during the summer or early fall. The underlying mechanisms warrant further study. Nonetheless, these patterns could help inform public health response to groups of viruses that tend to circulate around the same time.

During the COVID‐19 pandemic, while most respiratory viruses had reduced circulation in NYC, epidemic dynamics of individual viruses differed. Influenza viruses, human endemic coronaviruses, and RSV typically circulate during cold months (from late fall to early spring the next year in NYC). Thus, circulation of all three groups of viruses was in decline at the start of the first pandemic wave (i.e., March 2020). As a result, the full impact of the COVID‐19 pandemic on these viruses did not manifest until the following respiratory virus season (beginning in October 2020), during which significant case reductions were found. Influenza cases remained low while RSV began to surface in the spring of 2021 (Figure 1B). In addition to reduced contact among individuals due to social distancing and infection reduction due to mask‐wearing, the reduced importation of new virus strains from travelers may have also played a role in the observed outcomes for these viruses. In particular, for influenza, seeding of new A(H3N2) strains emerged globally has been shown to play an important role in starting new epidemics in North America.^17^ As such, given the likely high population susceptibility to these viruses after the skipped 2020–2021 season, it is important to monitor the circulation of these viruses (particularly influenza) in the population, severity of cases, concentration in geographical area and/or settings (e.g., congregate facilitates), and age groups in future years. This would enable the implementation of more timely infection mitigation measures (e.g., targeted messaging including vaccination promotion).

For PIV, HMPV, and RV, because their epidemic timing coincided with the first COVID‐19 pandemic wave in NYC (spring 2020), all three viruses saw the most dramatic case declines in the first few months of the pandemic. However, as NYC partially reopened in the summer of 2020, the epidemic trajectories of these viruses evolved differently, likely due to the differences in infection demographics. Both PIV and HMPV predominantly infect young children^18^, ^19^ and, to a lesser extent, older adults.^20^, ^21^ As such, their transmission was reduced to minimal levels in both key infection age groups when schools closed and intergenerational interactions (i.e., between grand‐children and grandparents) reduced during the pandemic. Interestingly, circulation of both viruses remained low after daycares and schools partially reopened, likely due to required preventive measures such as physical distancing and mask wearing in schools. This low transmission in young children may have in part reduced the risk of transmission to and among older adults. Future work using more detailed data (e.g., household data) may further examine the importance of intergenerational transmission of these viruses and provide insights into infection prevention for older adults, for whom infections could lead to severe health outcomes.^20^

For RV, even though viral activities were also lower during the COVID‐19 period, case increases were observed during the summer of 2020 when NPIs were relaxed (Figure 1D). Similarly, continued transmission of RV was reported during and after the 2009 influenza A(H1N1) pandemic.^22^ Rhinovirus infections occur in most age groups, and infection of one serotype confers little immune protection against others.^23^, ^24^, ^25^ This wider infection demographics and the large breadth of RV serotypes (around 160 discovered by 2018^24^) likely facilitated its transmission locally in the population.

Our study has several limitations. First, the data analyzed here are a subset of all tests done in NYC (i.e., only those from laboratories using the expanded respiratory panel tests) and thus may not be fully representative of the entire population. Second, even though the selection criteria have not changed during the study period, underlying patient characteristics may differ among specimens tested before and during the COVID‐19 period, due to changing medical seeking behaviors in response to COVID‐19 (e.g., people with mild respiratory symptoms may be more likely to seek testing at the early stage of the pandemic due to concern of COVID‐19); this in turn may temporally change the composition of underlying sample population. Third, fewer specimens were tested each week during the early phase of the pandemic due to limited testing supplies and human resources; this reduced sampling likely increased model uncertainty. Fourth, the identified associations (Tables 1 and S2) were based on population‐level epidemic time series and do not imply any causal interactions between each included virus pairs. Future research at the individual level (e.g., frequency of co‐infection or subsequent infections by multiple viruses in the same individuals) is warranted to further examine the potential viral interactions reported here. Finally, although we found substantial case reductions during the pandemic for several non‐SARS‐CoV‐2 respiratory viruses, it is difficult to distinguish the impact due to the introduction and circulation of the SARS‐CoV‐2 virus in the population and that due to the NPIs. Long‐term viral surveillance post‐pandemic may allow further study on the interactions between SARS‐CoV‐2 and other respiratory viruses without the presence of NPIs and in turn better understanding of the impact of NPIs on each virus.

## AUTHOR CONTRIBUTIONS

**Haokun Yuan:** Formal analysis; investigation; methodology; validation; visualization. **Alice Yeung:** Data curation; investigation. **Wan Yang:** Conceptualization; funding acquisition; investigation; methodology; project administration; supervision; validation.

### PEER REVIEW

The peer review history for this article is available at https://publons.com/publon/10.1111/irv.12976.

## Supporting information


**Figure S1.** Number of samples tested for respiratory viruses during each week over the study period. The vertical black line indicates the timing of COVID‐19 pandemic.

**Figure S2.** Comparison of the adjusted incidence used in this study and percent positivity (i.e., test positive rate) for each virus.

**Figure S3.** SARIMA and SARIMAX model validation for adenovirus (Adv; A), respiratory syncytial virus (RSV; B), human metapneumovirus (HMPV; C), rhinovirus (RV; D), and influenza virus (IV; E). SARIMA and SARIMAX models for each virus were first trained using incidence data from Week 40 of 2015 to Week 39 of 2019 and then used to generate out‐of‐fit estimates (i.e., prediction) of incidence from Week 40 of 2019 to Week 9 of 2020. Crosses (‘x’) show scaled weekly incidence. Blue lines (mean) and shaded areas (95% confidence intervals) show model fit (solid lines) and out‐of‐fit estimates (dashed lines) using the SARIMA models; red lines (mean) and shaded areas (95% confidence intervals) show model fit (solid lines) and out‐of‐fit estimates (dashed lines) using the SARIMAX models.

**Figure S4.** SARIMA and SARIMAX model validation for human endemic coronaviruses: CoV‐NL63 (A), CoV‐HKU (B), CoV‐OC43 (C), and CoV‐229E (D). SARIMA and SARIMAX models for each virus were first trained using incidence data from Week 40 of 2015 to Week 39 of 2019 and then used to generate out‐of‐fit estimates (i.e., prediction) of incidence from Week 40 of 2019 to Week 9 of 2020. Crosses (‘x’) show scaled weekly incidence. Blue lines (mean) and shaded areas (95% confidence intervals) show model fit (solid lines) and out‐of‐fit estimates (dashed lines) using the SARIMA models; red lines (mean) and shaded areas (95% confidence intervals) show model fit (solid lines) and out‐of‐fit estimates (dashed lines) using the SARIMAX models.

**Figure S5.** SARIMA and SARIMAX model validation for parainfluenza viruses: PIV‐1 (A), PIV‐2 (B), PIV‐3 (C), and PIV‐4 (D). SARIMA and SARIMAX models for each virus were first trained using incidence data from Week 40 of 2015 to Week 39 of 2019 and then used to generate out‐of‐fit estimates (i.e., prediction) of incidence from Week 40 of 2019 to Week 9 of 2020. Crosses (‘x’) show scaled weekly incidence. Blue lines (mean) and shaded areas (95% confidence intervals) show model fit (solid lines) and out‐of‐fit estimates (dashed lines) using the SARIMA models; red lines (mean) and shaded areas (95% confidence intervals) show model fit (solid lines) and out‐of‐fit estimates (dashed lines) using the SARIMAX models.

**Table S1.** Parameters (p, d, q) used in SARMIA and SARIMAX models for each virus. Note that, for the seasonal component, we applied 1‐degree seasonal differencing and did not include any seasonal autoregressive or moving average terms (i.e., setting P = 0, D = 1, and Q = 0 for the corresponding seasonal regression parameters) for all models.
**Table S2.** Estimated strengths of virus interactions. For each virus, interacting viruses were identified first by the elastic net regression and further by forward stepwise selection into the final SARIMAX model. Each column shows results for the final SARIMAX model for each of the thirteen viruses included here: non‐empty cells indicate viruses in the corresponding rows (see row names) were included and numbers show estimated coefficients in the SARIMAX model (those with * indicate a significant interaction, i.e., p value < 0.05); empty cells indicate the corresponding viruses were not included in the SARIMAX model as interacting viruses. The directions and strengths of interactions are color‐coded: warm colors represent positive interactions, cold colors represent negative interactions, and darker shades indicate stronger interactions. For comparison, the estimates shown here are computed using normalized time series (with a zero mean and standard deviation of 1) such that estimated strengths of interaction are on the same scale for all viruses. For instance, for adenovirus (Adv), five viruses (CoV‐NL63, HMPV, RV, RSV and IV) were identified as its interacting viruses; the estimated strengths were strongest for RSV (−0.45), followed by IV (0.39), CoV‐NL63 (0.2) and RV (0.2), and HMPV (−0.19).
**Table S3.** Model performance during pre‐COVID testing period (Oct 2019‐Feb 2020). Virus interactions were the exogenous variables included in the SARIMAX model. Model performance was measured by relative RMSE and the difference in relative RMSE between the two models is shown in the parentheses.

## Data Availability

The virus surveillance data were used with permission under a Data Use and Nondisclosure Agreement between the New York City Department of Health and Mental Hygiene and Columbia University.
