# Supplementary figures and images for: Interactions among common non‐SARS‐CoV‐2 respiratory viruses and influence of the COVID‐19 pandemic on their circulation in New York City
Source: Influenza Other Respir Viruses. 2022 Mar 12;16(4):653–61. doi: 10.1111/irv.12976 (PMC9111828; doi:10.1111/irv.12976)

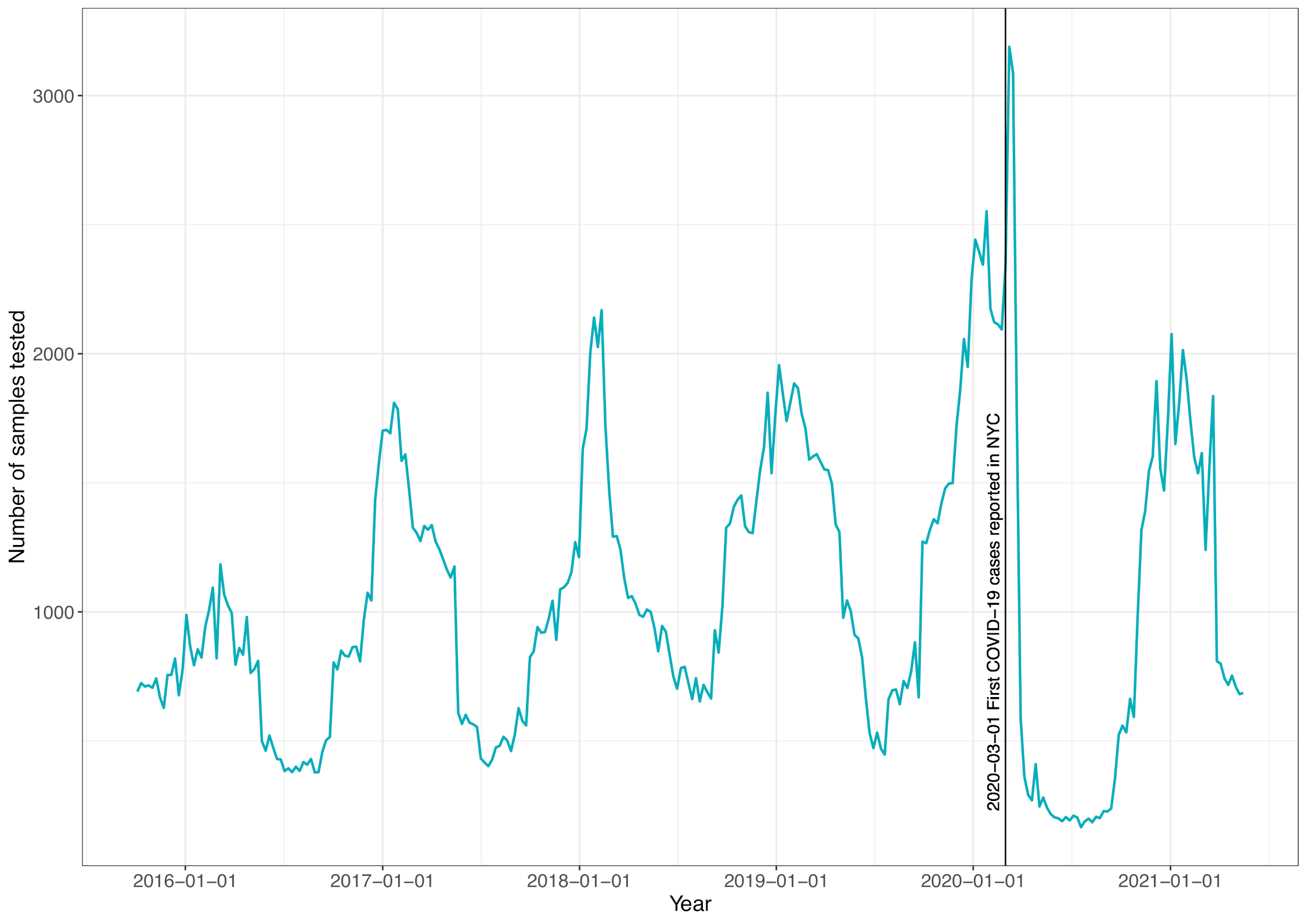

Supplement: Supplementary file 1 — Figure S1. Number of samples tested for respiratory viruses during each week over the study period. The vertical black line indicates the timing of COVID‐19 pandemic. [file IRV-16-653-s001.tif]

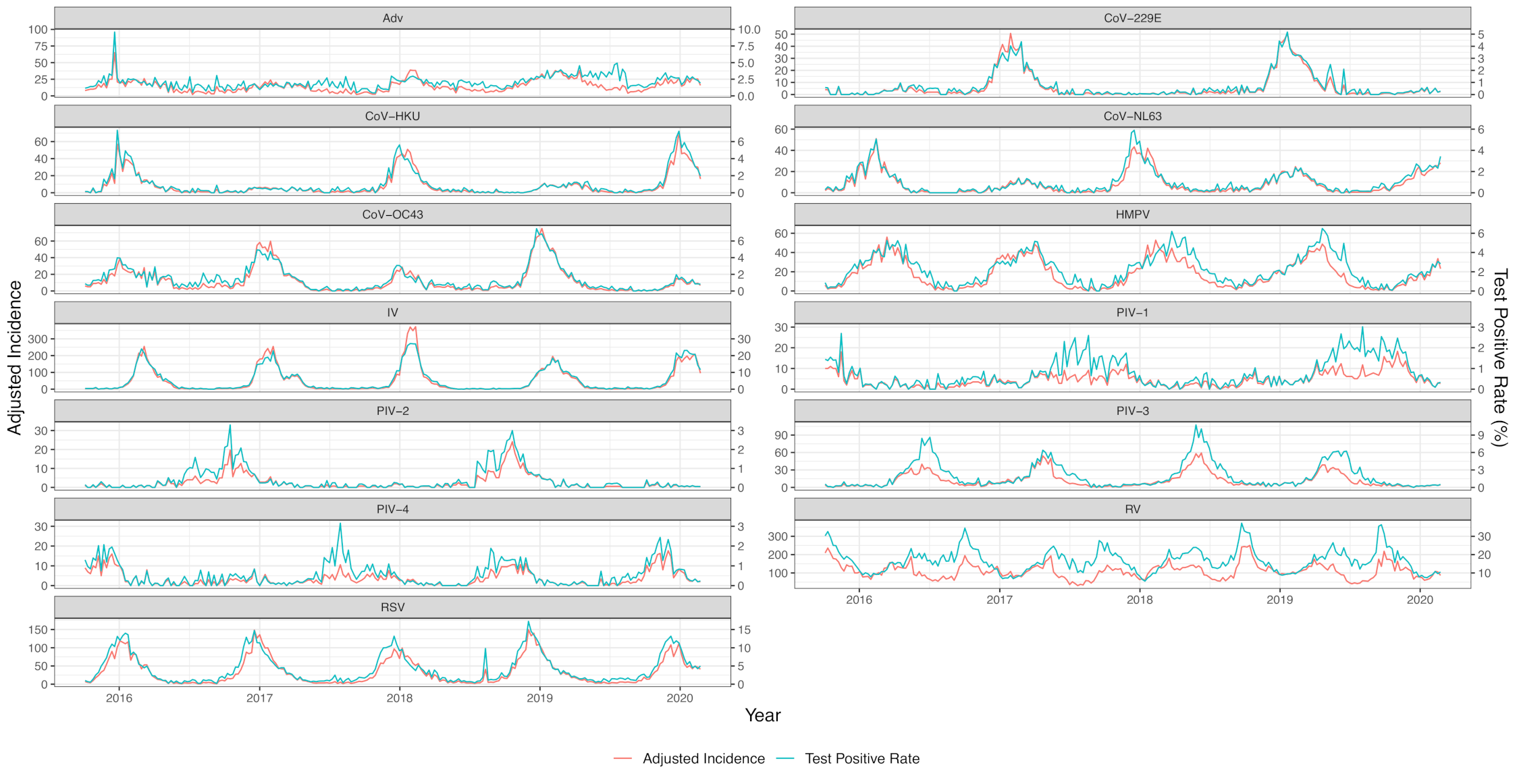

Supplement: Supplementary file 2 — Figure S2. Comparison of the adjusted incidence used in this study and percent positivity (i.e., test positive rate) for each virus. [file IRV-16-653-s003.tif]

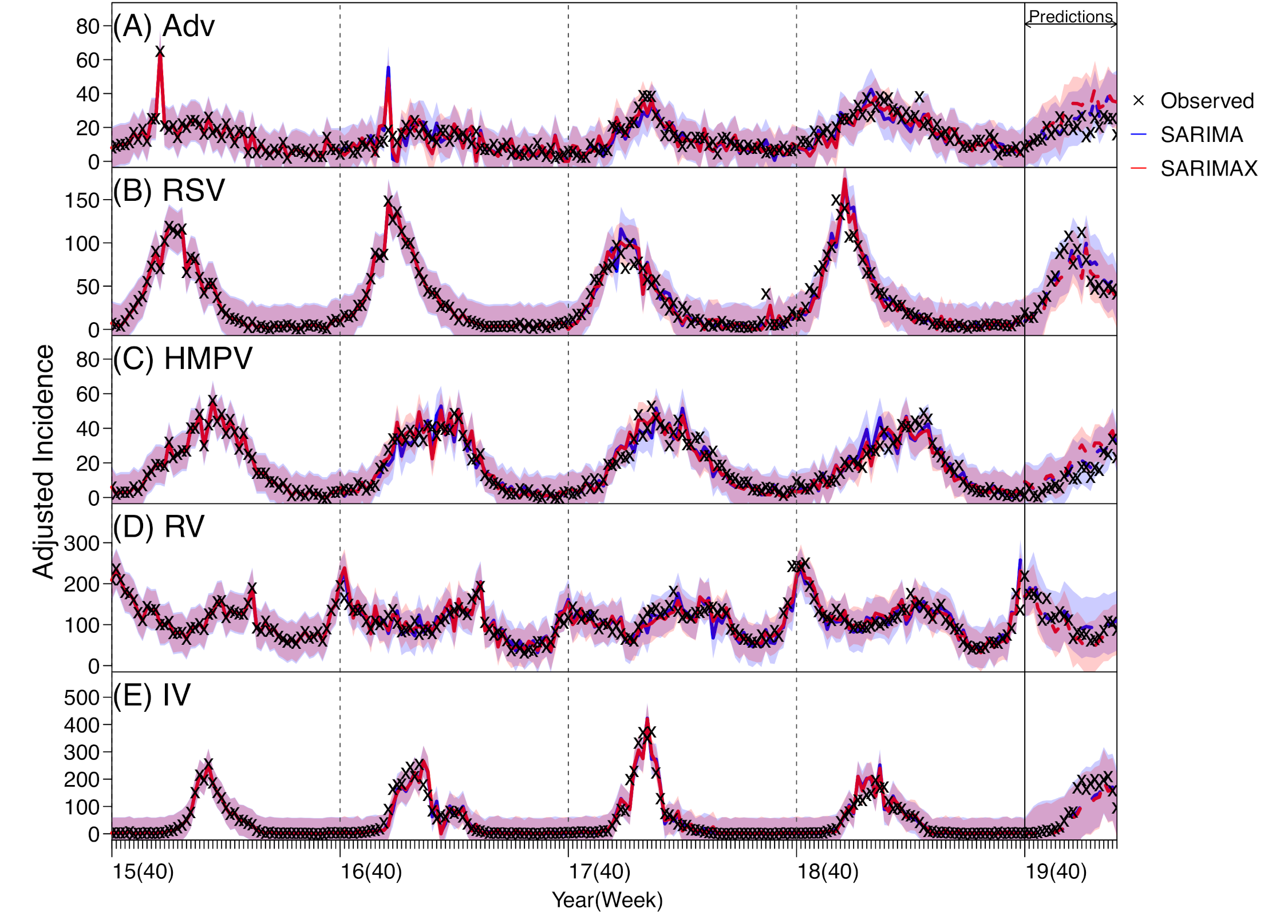

Supplement: Supplementary file 3 — Figure S3. SARIMA and SARIMAX model validation for adenovirus (Adv; A), respiratory syncytial virus (RSV; B), human metapneumovirus (HMPV; C), rhinovirus (RV; D), and influenza virus (IV; E). SARIMA and SARIMAX models for each virus were first trained using incidence data from Week 40 of 2015 to Week 39 of 2019 and then used to generate out‐of‐fit estimates (i.e., prediction) of incidence from Week 40 of 2019 to Week 9 of 2020. Crosses (‘x’) show scaled weekly incidence. Blue lines (mean) and shaded areas (95% confidence intervals) show model fit (solid lines) and out‐of‐fit estimates (dashed lines) using the SARIMA models; red lines (mean) and shaded areas (95% confidence intervals) show model fit (solid lines) and out‐of‐fit estimates (dashed lines) using the SARIMAX models. [file IRV-16-653-s004.tif]

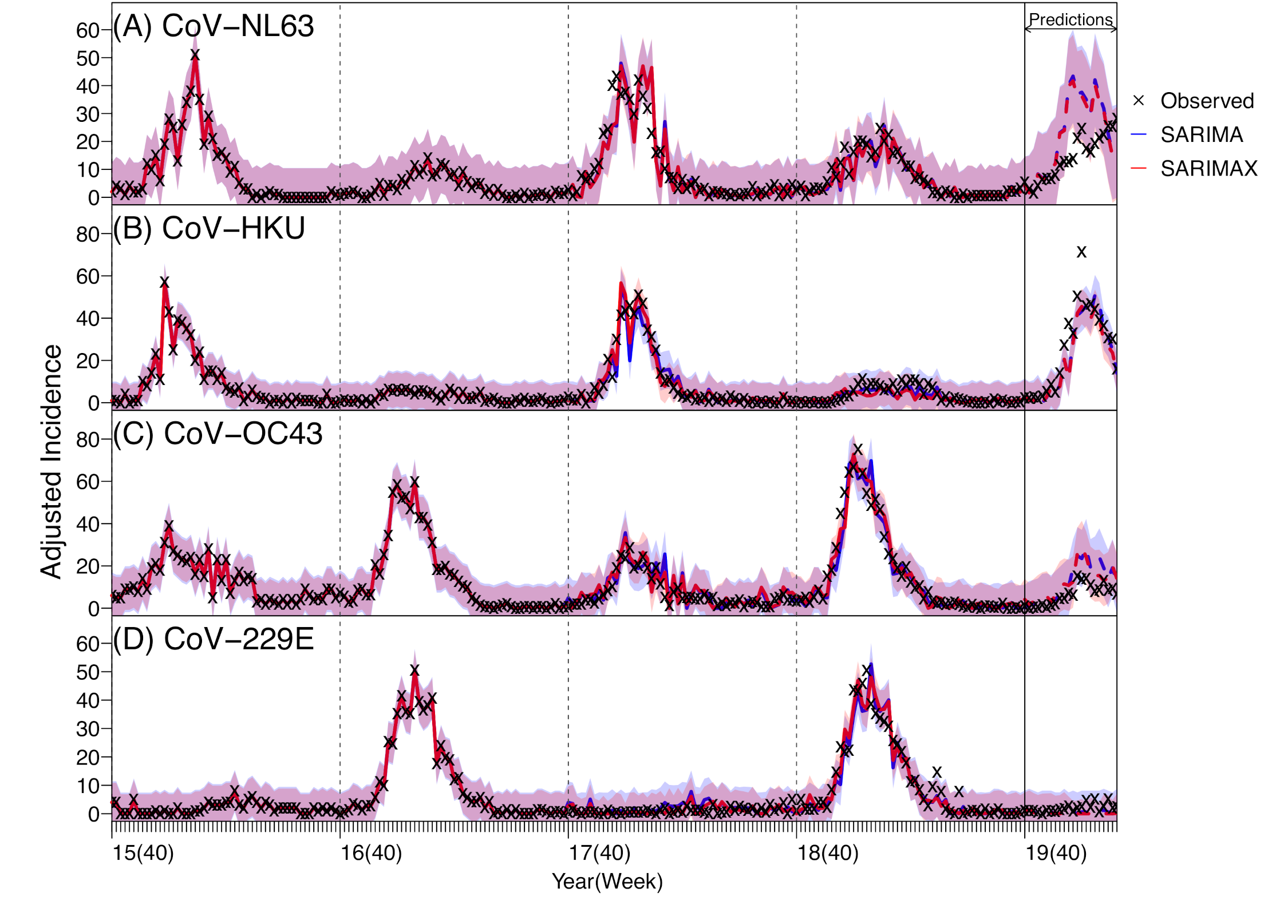

Supplement: Supplementary file 4 — Figure S4. SARIMA and SARIMAX model validation for human endemic coronaviruses: CoV‐NL63 (A), CoV‐HKU (B), CoV‐OC43 (C), and CoV‐229E (D). SARIMA and SARIMAX models for each virus were first trained using incidence data from Week 40 of 2015 to Week 39 of 2019 and then used to generate out‐of‐fit estimates (i.e., prediction) of incidence from Week 40 of 2019 to Week 9 of 2020. Crosses (‘x’) show scaled weekly incidence. Blue lines (mean) and shaded areas (95% confidence intervals) show model fit (solid lines) and out‐of‐fit estimates (dashed lines) using the SARIMA models; red lines (mean) and shaded areas (95% confidence intervals) show model fit (solid lines) and out‐of‐fit estimates (dashed lines) using the SARIMAX models. [file IRV-16-653-s002.tif]

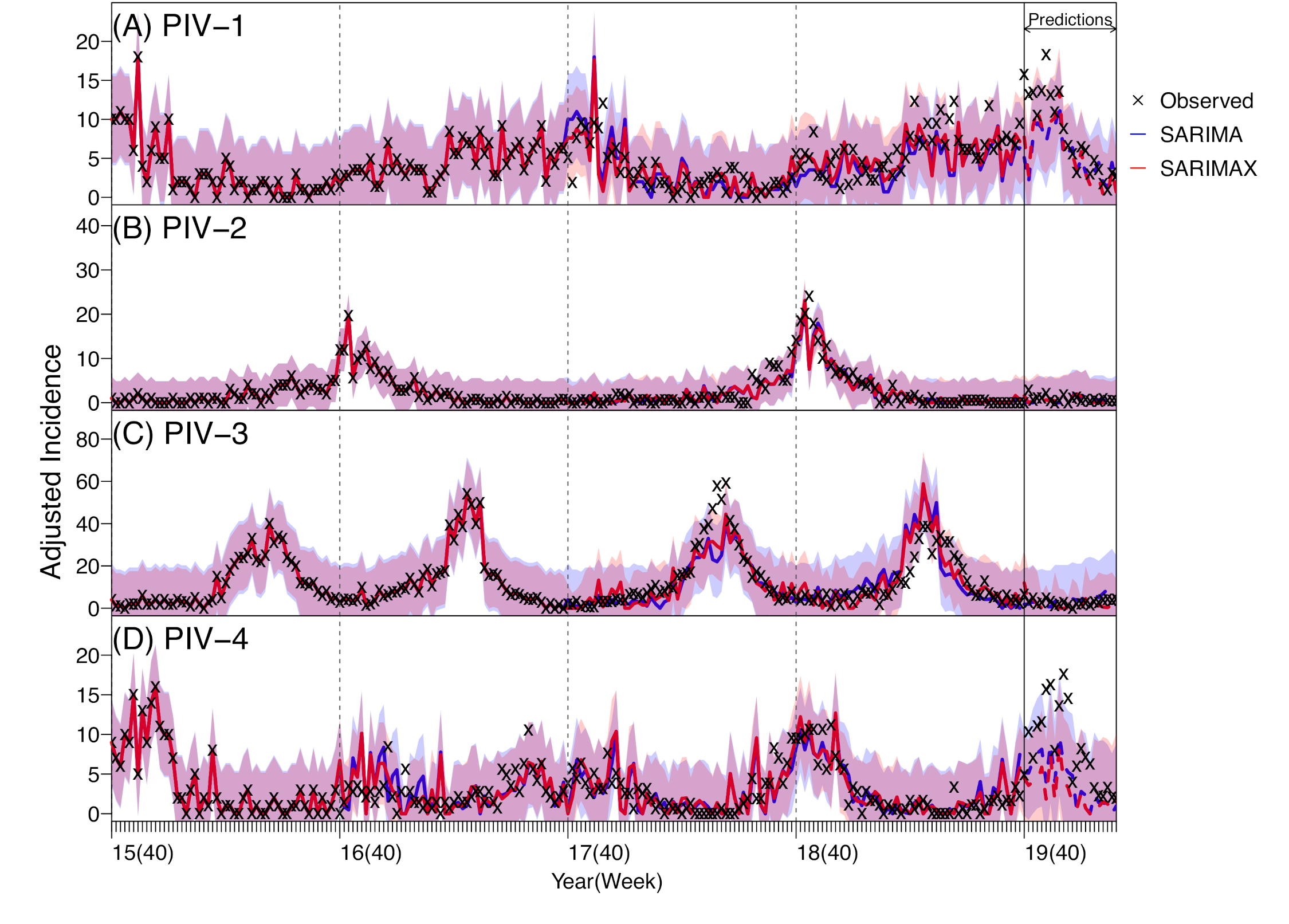

Supplement: Supplementary file 5 — Figure S5. SARIMA and SARIMAX model validation for parainfluenza viruses: PIV‐1 (A), PIV‐2 (B), PIV‐3 (C), and PIV‐4 (D). SARIMA and SARIMAX models for each virus were first trained using incidence data from Week 40 of 2015 to Week 39 of 2019 and then used to generate out‐of‐fit estimates (i.e., prediction) of incidence from Week 40 of 2019 to Week 9 of 2020. Crosses (‘x’) show scaled weekly incidence. Blue lines (mean) and shaded areas (95% confidence intervals) show model fit (solid lines) and out‐of‐fit estimates (dashed lines) using the SARIMA models; red lines (mean) and shaded areas (95% confidence intervals) show model fit (solid lines) and out‐of‐fit estimates (dashed lines) using the SARIMAX models. [file IRV-16-653-s005.tif]
