# Supplementary material for: Interactions among common non‐SARS‐CoV‐2 respiratory viruses and influence of the COVID‐19 pandemic on their circulation in New York City
Source: Influenza Other Respir Viruses. 2022 Mar 12;16(4):653–61. doi: 10.1111/irv.12976 (PMC9111828; doi:10.1111/irv.12976)
Supplement: Supplementary file 6 — Table S1. Parameters (p, d, q) used in SARMIA and SARIMAX models for each virus. Note that, for the seasonal component, we applied 1‐degree seasonal differencing and did not include any seasonal autoregressive or moving average terms (i.e., setting P = 0, D = 1, and Q = 0 for the corresponding seasonal regression parameters) for all models. Table S2. Estimated strengths of virus interactions. For each virus, interacting viruses were identified first by the elastic net regression and further by forward stepwise selection into the final SARIMAX model. Each column shows results for the final SARIMAX model for each of the thirteen viruses included here: non‐empty cells indicate viruses in the corresponding rows (see row names) were included and numbers show estimated coefficients in the SARIMAX model (those with * indicate a significant interaction, i.e., p value < 0.05); empty cells indicate the corresponding viruses were not included in the SARIMAX model as interacting viruses. The directions and strengths of interactions are color‐coded: warm colors represent positive interactions, cold colors represent negative interactions, and darker shades indicate stronger interactions. For comparison, the estimates shown here are computed using normalized time series (with a zero mean and standard deviation of 1) such that estimated strengths of interaction are on the same scale for all viruses. For instance, for adenovirus (Adv), five viruses (CoV‐NL63, HMPV, RV, RSV and IV) were identified as its interacting viruses; the estimated strengths were strongest for RSV (−0.45), followed by IV (0.39), CoV‐NL63 (0.2) and RV (0.2), and HMPV (−0.19). Table S3. Model performance during pre‐COVID testing period (Oct 2019‐Feb 2020). Virus interactions were the exogenous variables included in the SARIMAX model. Model performance was measured by relative RMSE and the difference in relative RMSE between the two models is shown in the parentheses. [file IRV-16-653-s006.docx]

**Supplemental Tables and Figures**

**Interactions among common non-SARS-CoV-2 respiratory viruses and influence of the COVID-19 pandemic on their circulation in New York City**

Haokun Yuan,^1^ Alice Yeung,^2^ Wan Yang^1^

^1^Department of Epidemiology, Mailman School of Public Health, Columbia University; ^2^Bureau of Communicable Disease, New York City Department of Health and Mental Hygiene

This document includes supplemental Tables S1-S3 and Figures S1-S5.

**Table S1.** Parameters (*p*, *d*, *q*) used in SARMIA and SARIMAX models for each virus. Note that, for the seasonal component, we applied 1-degree seasonal differencing and did not include any seasonal autoregressive or moving average terms (i.e., setting *P*=0, *D*=1, and *Q*=0 for the corresponding seasonal regression parameters) for all models.

| Respiratory Virus (Sub)type | | SARIMA/SARIMAX parameters | | |
| --- | --- | --- | --- | --- |
|  |  | *p* | *d* | *q* |
| Adenovirus  (Adv) | | 2 | 0 | 0 |
| Human endemic coronavirus (CoV) | CoV-NL63 | 1 | 0 | 0 |
|  | CoV-HKU | 2 | 0 | 0 |
|  | CoV-OC43 | 3 | 0 | 0 |
|  | CoV-229E | 2 | 0 | 0 |
| Human Metapneumovirus (HMPV) | | 2 | 0 | 0 |
| Rhinovirus (RV) | | 3 | 0 | 0 |
| Parainfluenza (PIV) | PIV-1 | 0 | 0 | 0 |
|  | PIV-2 | 2 | 0 | 0 |
|  | PIV-3 | 0 | 0 | 0 |
|  | PIV-4 | 3 | 0 | 0 |
| Respiratory Syncytial Virus (RSV) | | 3 | 0 | 0 |
| Influenza virus (IV) | | 3 | 0 | 0 |

**Table S2.** Estimated strengths of virus interactions. For each virus, interacting viruses were identified first by the elastic net regression and further by forward stepwise selection into the final SARIMAX model. Each column shows results for the final SARIMAX model for each of the thirteen viruses included here: non-empty cells indicate viruses in the corresponding rows (see row names) were included and numbers show estimated coefficients in the SARIMAX model (those with * indicate a significant interaction, i.e., p value < 0.05); empty cells indicate the corresponding viruses were not included in the SARIMAX model as interacting viruses. The directions and strengths of interactions are color-coded: warm colors represent positive interactions, cold colors represent negative interactions, and darker shades indicate stronger interactions. For comparison, the estimates shown here are computed using normalized time series (with a zero mean and standard deviation of 1) such that estimated strengths of interaction are on the same scale for all viruses. For instance, for adenovirus (Adv), five viruses (CoV-NL63, HMPV, RV, RSV and IV) were identified as its interacting viruses; the estimated strengths were strongest for RSV (-0.45), followed by IV (0.39), CoV-NL63 (0.2) and RV (0.2), and HMPV (-0.19).

**
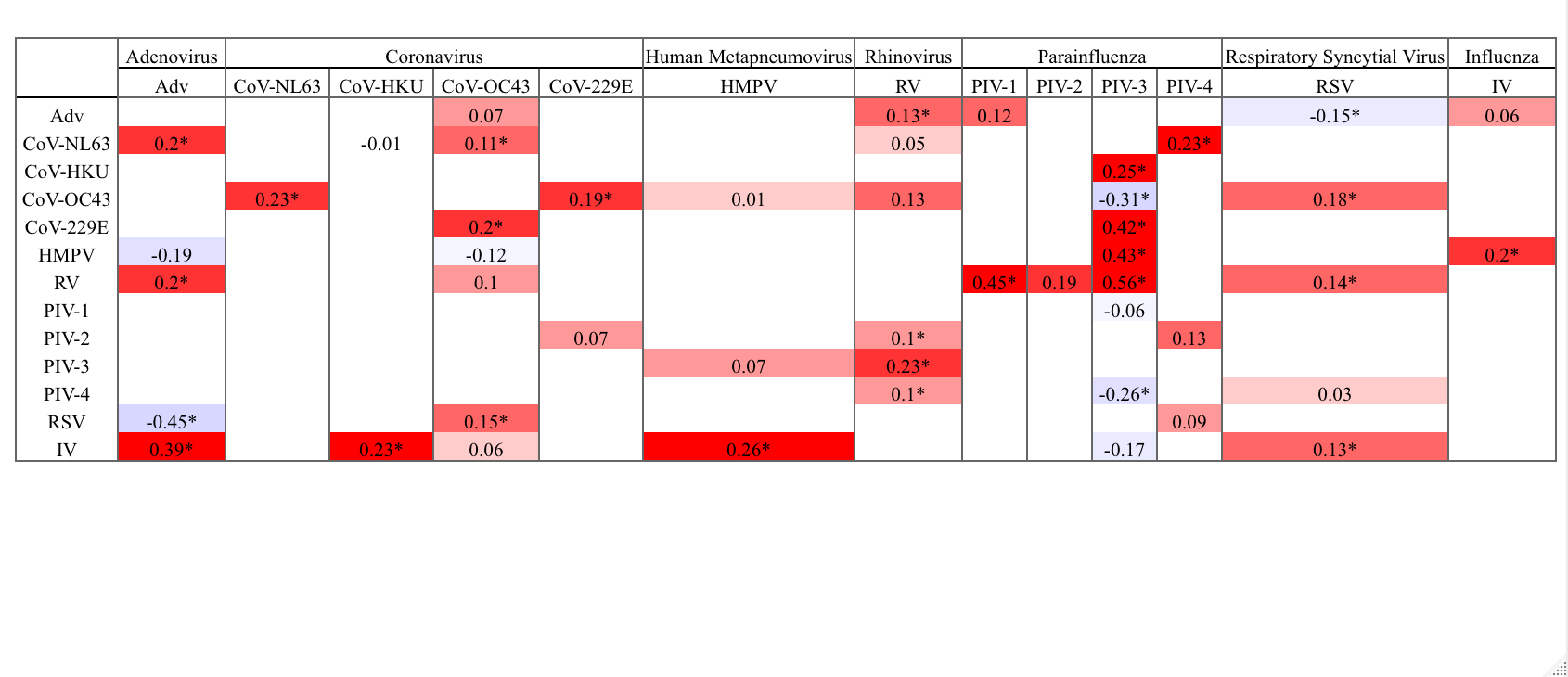
**

**Table S3.** Model performance during pre-COVID testing period (Oct 2019-Feb 2020). Virus interactions were the exogenous variables included in the SARIMAX model. Model performance was measured by relative RMSE and the difference in relative RMSE between the two models is shown in the parentheses.

|  | | Virus Interactions | Relative RMSE  (Testing period) | |
| --- | --- | --- | --- | --- |
|  |  |  | SARIMA | SARIMAX |
| Adenovirus  (Adv) | | IV, RSV, CoV-NL63, RV, HMPV | 0.69 | 0.87  (26.09%) |
| Human endemic coronavirus (CoV) | CoV-NL63 | CoV-OC43 | 1.88 | 1.78  (-5.32%) |
|  | CoV-HKU | IV, CoV-NL63 | 1.3 | 1.27  (-2.31%) |
|  | CoV-OC43 | CoV-229E, RV, IV, CoV-NL63, HMPV, RSV, Adv | 0.76 | 0.68  (-10.53%) |
|  | CoV-229E | CoV-OC43, PIV-2 | 0.38 | 0.66  (73.68%) |
| Human Metapneumovirus  (HMPV) | | IV, PIV-3, CoV-OC43 | 0.38 | 0.61  (60.53%) |
| Rhinovirus  (RV) | | PIV-2, PIV-3, Adv, PIV-4, CoV-OC43, CoV-NL63 | 0.27 | 0.26  (-3.7%) |
| Parainfluenza (PIV) | PIV-1 | RV, Adv | 1.25 | 1.08  (-13.6%) |
|  | PIV-2 | RV | 0.47 | 0.48  (2.13%) |
|  | PIV-3 | RV, PIV-4, CoV-229E, CoV-HKU, HMPV, IV, CoV-OC43, PIV-1 | 0.36 | 0.75  (108.33%) |
|  | PIV-4 | CoV-NL63, PIV-2, RSV | 1.89 | 2.35  (24.34%) |
| Respiratory Syncytial Virus  (RSV) | | CoV-OC43, Adv, RV, IV, PIV-4 | 0.82 | 0.69  (-15.85 %) |
| Influenza  (IV) | | HMPV, Adv | 0.9 | 1.01  (12.22%) |
